# Combined effects of nitrate and antimicrobial compounds on *in vitro* subgingival biofilms

**DOI:** 10.1038/s41598-026-36588-x

**Published:** 2026-01-30

**Authors:** Siobhan P. Moran, Maria Nadal-Ruiz, Alex Mira, Ana Serrano-Valcarce, Mercedes Mompeán, Miglė Žiemytė, Andrés López-Roldán, Fiona L. Henriquez, Mia C. Burleigh, Bob T. Rosier

**Affiliations:** 1 School of Health and Life Sciences, University of The West of Scotland, Blantyre, Scotland; 2 Department of Health and Genomics, Center for Advanced Research in Public Health, FISABIO Foundation, Avenida de Catalunya 21, Valencia, 46020 Spain; 3 Department of Periodontology, University of Valencia, Valencia, Spain

**Keywords:** Diseases, Medical research, Microbiology

## Abstract

Chlorhexidine and antibiotics are commonly used as adjunct treatments for periodontitis. However, these antimicrobials can lead to microbial resistance and chlorhexidine can impair health-associated nitrate (NO_3_^-^) metabolism. We tested the effect of chlorhexidine (0.002%), metronidazole (16 µg/ml) and amoxicillin (0.7 µg/ml), with and without 8 mM NO_3_^-^, on the bacterial composition and NO_3_^-^ metabolism of subgingival plaque samples from 12 periodontitis patients grown in vitro for 8 h. The low sublethal concentrations of amoxicillin and chlorhexidine significantly inhibited microbial growth and impaired NO_3_^-^ reduction, whereas the physiological concentration of metronidazole did not. A lower subgingival microbial dysbiosis index (SMDI) was found in the NO_3_^-^ condition compared with amoxicillin alone and chlorhexidine with or without NO_3_^-^ (*p* < 0.05). The SMDI of the metronidazole conditions was also significantly lower than in those with chlorhexidine (*p* < 0.05). Moreover, NO_3_^-^ alone or combined with metronidazole appeared to increase *Neisseria* spp. and *Aggregatibacter* spp., whilst disease-associated changes were found in the chlorhexidine and amoxicillin conditions. Adding NO_3_^-^ to metronidazole led to health-associated changes compared with metronidazole alone. In conclusion, low levels of amoxicillin and chlorhexidine limited microbial growth, impaired NO_3_^-^ metabolism and were linked to disease-associated microbial profiles. A dual treatment of metronidazole + NO_3_^-^ should be further investigated in clinical studies.

## Introduction

Periodontitis is a chronic oral inflammatory disease characterised by a build-up of dental plaque (bacterial biofilm) and progressive deterioration of the periodontium (supporting structures and tissues of the teeth)^1^. Worldwide, periodontitis affects ≈ 796 million people, with an estimated 11% living with severe periodontitis^1,2^. The disease causes a shift in the oral microbiota towards disease-associated bacterial species and functions, known as dysbiosis^3,4^. The hosts inflammatory response, combined with bacterial by-products, causes deterioration of periodontal structures^4,5^, which can lead to bleeding and tooth loss, ultimately resulting in impaired speech and mastication^5^. Additionally, the impact of periodontitis extends beyond the oral cavity, increasing the risk of several systemic conditions, such as cardiovascular disease, type II diabetes and neurodegenerative conditions such as Alzheimer’s disease^6,7^.

Mechanical plaque removal is the first line of treatment for periodontitis. Additionally, depending on disease severity and treatment protocols, adjunctive therapies may include systemic antibiotics (usually metronidazole and amoxicillin) and antiseptics such as chlorhexidine^8,9^. Chlorhexidine is an antimicrobial agent found in some commercially available mouthwashes^10^. The main aim of periodontal treatment is to eradicate disease-causing bacteria and reduce the hosts inflammatory response^11,12^. Unfortunately, broad-spectrum antibiotics and antiseptic mouthwashes can kill both beneficial and harmful bacteria, sometimes exacerbating dysbiosis^10,12^. For example, Bescos et al.^13^, showed that a 7-day chlorhexidine treatment in vivo lowered microbial diversity, increased salivary lactate, and decreased pH, contributing to dental caries risk. Systemic antibiotics, in contrast, must pass through the bloodstream and can negatively affect the gut microbiota^12^. Additionally, as with antibiotics, chlorhexidine has been linked to antimicrobial resistance (AMR), as bacteria deeper in the biofilm may encounter sub-lethal concentrations, reducing killing efficiency^10,14^. The impact of amoxicillin and metronidazole on oral microbiota composition remains unclear and should be explored to determine potential contributions to dysbiosis.

In contrast, NO_3_^−^ has been proposed as an alternative treatment for periodontitis^15,16^. NO_3_^−^ is a prebiotic that is naturally abundant in leafy greens (e.g., lettuce and spinach) and root vegetables such as beetroot and radishes^16,17^. After consumption, NO_3_^−^ enters the gastrointestinal tract, is absorbed into the bloodstream and concentrated by the salivary glands. In the oral cavity, nitrate reducing bacteria (NRB) (e.g., *Rothia* and *Neisseria* species) convert NO_3_^−^ to NO_2_^−^, which is then swallowed and further reduced to nitric oxide (NO) in the stomach and other body tissue after entering the systemic circulation^18,19^. This process is known as the NO_3_^−^ - NO_2_^−^ - NO pathway, which contributes to systemic NO availability^16,17^. NO is a multi-functional signalling molecule that supports host defence, glucose homeostasis, smooth muscle contraction and mitochondrial function^20–23^. Its vasodilatory effects also enhance blood flow, indirectly benefiting neuronal, metabolic and cardiovascular health^19,24^. Notably, two weeks of chlorhexidine use wash shown to impair NO_3_^−^ reduction, lower plasma NO_2_^−^ concentrations, and slightly increased blood pressure compared to a placebo mouthrinse, demonstrating that antimicrobial compounds in the oral cavity can have systemic implications^13,25^.

Importantly, NO is also beneficial for oral health, as the molecule has antimicrobial properties against anaerobic bacteria associated with periodontitis^26^. Moreover, NO can signal to host cells and produce anti-inflammatory effects by activating the cGMP pathway^27^. In short, NO_3_^−^ can help to restore eubiosis in the oral cavity, by decreasing the proportion of disease-associated bacterial species and increasing health-associated NRB (e.g., *Rothia* and *Neisseria*)^16,28^.

To the best of our knowledge, no studies have analysed the effects of chlorhexidine and common antibiotics on the bacterial composition of subgingival plaque samples. Additionally, it is unknown how common antibiotics affect the NO_3_^−^ reduction capacity of the oral microbiota. In this current work, we aim to determine the effects of two antibiotics (amoxicillin and metronidazole) and chlorhexidine, in the absence or presence of NO_3_^−^, on the bacterial composition of subgingival plaque. Additionally, we aim to determine how these antimicrobial compounds affect NO_3_^−^ metabolism of the subgingival communities. Understanding these effects is essential for guiding more efficient, microbiome-friendly treatment strategies that preserve beneficial functions of the oral ecosystem while effectively managing the disease.

## Materials and methods

### Patient inclusion and sampling procedure

Twelve adult patients (3 males, 9 females, average age 57.5 years ± 9.7 years) with periodontitis, each having at least five teeth in every quadrant, were recruited at the Lluis Alcanyis Foundation, dental clinic of the University of Valencia (Valencia, Spain). All participants signed an informed consent prior to sample donation and the study protocol was approved by the Ethics Committee of the University of Valencia (Spain), with the reference 2023-ODON-3,003,121. This study was carried out according to the relevant guidelines and regulations of the Declaration of Helsinki. The diagnosis of periodontitis was determined in accordance with the guidelines of The American Academy of Periodontology; (AAP)^29^. Exclusion criteria were (i) the use of systemic antibiotics or antiseptic mouthwash in the last month, (ii) active systemic infection, (iii) periodontal treatment in the previous 6 months, (iv) and pregnancy.

Subgingival plaque was collected from the periodontal pocket at five different periodontitis sites (≥ 4 mm PPD and ≥ 1 mm CAL) in each patient using 13 sterile paper points. Paper points were transferred to a 2 mL tube containing reduced transport medium^30^ and stored at ~ 4 °C. The samples were transported to the laboratory and tested within 12–20 h.

### Real-time in vitro biofilm growth of periodontal plaque

Real-time *in vitro* biofilm growth was assessed using the xCELLigence RTCA single plate system (ACEA Biosciences, San Diego, California, USA). This system detects changes in electrical impedance caused by bacterial adhesion and biofilm formation on electrode-containing wells, enabling continuous, label-free quantification of biofilm development^31^. The amount of biofilm growth is measured and expressed as “Cell Index” values, which correlate with total biofilm mass^32^.

Baseline cell-sensor impedance measurements were performed using 100 µl as described in the manufacturer’s instructions. For this, a 1:1 mixture of Brain Heart Infusion (BHI) medium (Oxoid, Termo Fisher Scientific, Waltham, Massachusetts, USA) and BHI without glucose (Condalab, Torrejón de Ardóz, Madrid, Spain) was used, limiting sugar availability and thus acidification, and was supplemented to obtain the following treatment conditions: control, NO_3_^−^ alone, metronidazole alone, metronidazole + NO_3_^−^, amoxicillin alone, amoxicillin + NO_3_^−^, chlorhexidine alone, and chlorhexidine + NO_3_^−^ (all Sigma-Aldrich, St. Louis, Missouri, USA). All these conditions were also supplemented with 5 mg/L hemin and 1 mg/L menadione (Sigma-Aldrich, St. Louis, Missouri, USA). Three baseline measurements of the control medium were performed in a 96-well plate with an integrated microelectronic cell sensor array (E-plate 96, ACEA Biosciences, San Diego, California, USA) at 3-min intervals. After this, another 50 µl of the medium was added.

Finally, 100 µl of BHI mixture medium containing periodontal plaque as the inoculum was added to each treatment condition. For each donor, the same bacterial suspension was used in all treatments to control inter-individual variability. This bacterial inoculum was prepared by vortexing the paper points for 30 s, then transferring 100 µl of the sample along with the paper points to a new tube, which was stored at −80 °C until sequencing to determine the initial bacterial composition. The procedure continued by centrifuging the tube containing the periodontal plaque sample (5 min, 2415 g), discarding the supernatant (transport medium), and re-suspending the bacterial pellet in 950 µl of BHI mixture medium supplemented with 5 mg/L hemin and 1 mg/L menadione (all Sigma-Aldrich, St. Louis, Missouri, USA). For the final concentrations of metronidazole, amoxicillin and chlorhexidine, the gingival crevicular fluid (GCF) levels of metronidazole were used (16 µg/ml), but GCF levels of amoxicillin (8 µg/ml) and commonly studied levels of chlorhexidine (0.02%)^33–35^ were bactericidal to subgingival biofilms during preliminary experiments. Therefore, these compounds were diluted 1:12, yielding final concentration of 0.0017% chlorhexidine and 0.67 µg/ml amoxicillin, which provided a balance between antimicrobial activity and bacterial growth, allowing to observe shifts in bacteria and microbial metabolism in the presence of these compounds. These low concentrations remain biologically relevant, as antimicrobials in GCF are substantially diluted within subgingival biofilms due to limited penetration, while still affecting bacterial metabolism and community composition^10,36,37^.

All plates were sealed with adhesive aluminum foil (VWR, Radnor, Pennsylvania, USA) to prevent oxygen diffusion, creating conditions that allow the growth of strict anaerobic bacteria, without requiring a fully anaerobic jar^31^. The plates were then incubated at 37 °C for 8 h, with cell impendence measurements recorded every 10 min. All experiments were performed without agitation, and anaerobic conditions were promoted by sealing the wells with adhesive aluminium foil, a method previously shown to support the growth of strictly anaerobic bacteria and the effect of different treatment conditions on their abundance after 5–12 h of incubation^16,31,38^. Supernatants, as well as the corresponding biofilms collected through resuspension using RNA later (Invitrogen, Waltham, Massachusetts, USA), were collected from the 96-well plate after the final cell-sensor impedance measurement. All supernatants and biofilms were stored at − 20 °C and − 80 °C, respectively.

### Determination of pH, nitrate, and nitrite concentrations

Supernatant collected from the *in vitro* biofilms was used to determine the pH levels and the concentration of NO_3_^−^ and NO_2_^−^ using the RQflex 10 Reflectoquant reflectometer (Merck Milipore, Burlington, Massachusetts, USA), following Rosier et al.^16^,. The supernatant was applied undiluted to pH test strips (reference no. 1169960001) and, either undiluted or diluted at least 5-fold in demineralized water, to the NO_3_^−^ (reference no. 1169710001) and NO_2_^−^ (reference no. 1169730001) test strips. For this, 15 µl of supernatant was pipetted on each of the two reactive patches of a test strip, excess liquid was removed by tipping the side of the strip on a tissue, and the strip was incubated according to the manufacturer’s instructions.

### Bacterial DNA extraction

DNA was extracted from biofilms and initial inoculum using the MasterPure Complete DNA and RNA purification Kit (Epicentre Biotechnology, Madison, Wisconsin, USA) following the manufacturer’s instructions, with the addition of lysozyme as described by Belda-Ferre et al.^39^ and Rosier et al.^16^,. The biofilms were prepared for extraction by centrifuging (10 min, 15870 g 4 °C), discarding the supernatant, washing the pellet with 1 ml PBS, re-centrifuging (10 min, 15870 g, 4 °C) and discarding the supernatant before addition of the lysis buffer (Epicentre Biotechnology). Moreover, the initial inocula were prepared for extraction by discarding paper points, centrifuging the plaque sample (10 min, 15870 g, 4 °C), discarding the supernatant (transport medium), washing the pellet with 1 ml PBS, re-centrifuging (10 min, 15870 g, 4 °C) and discarding the supernatant before addition of the lysis buffer. The isolated DNA was resuspended in 32 µL H20 miliQ and stored at − 80 °C for Illumina sequencing of the 16 S rRNA gene. Total DNA extracted from each biofilm sample was used an approximate measure of the amount of biofilm mass.

### Biofilm composition determined by 16S rRNA sequencing and read processing

DNA from 8 h *in vitro* biofilms, as well as the initial inocula, was sequenced to determine the bacterial composition.

For sequencing, an Illumina amplicon library was performed following the 16 S rRNA gene Metagenomic Sequencing Library Preparation Illumina protocol (Part #15044223 Rev.A). The primer sequences used in this protocol were: *Illumina_16S_341F* (TCGTCGGCAGCGTCAGATGTGTATAAGAGACAGCCTACGGGNGGCWGCAG) and *Illumina_16S_80v5R* (GTCTCGTGGGCTCGGAGATGTGTATAAGAGACAGGACTACHVGGGTATCTAATCC) which target the 16 S V3 and V4 region. Following amplification, cDNA was sequenced by FISABIO-Public Health Sequencing and Bioinformatics Service with an Illumina MiSeq Sequencer according to manufacturer’s instructions using the 2 × 300 base paired-ends protocol.

### Taxonomic classification

The DADA2 pipeline (v1.8) in R was used to process the paired-end FASTQ files. The forward and reverse reads were trimmed, removing the primer sequences and low-quality bases at the end of the reads through end-trimming. Reads with any ambiguous N base or exceeding 5 expected errors were also discarded. The forward and reverse pairs were combined, with a minimum overlap of 12 bases and a maximum mismatch of 1 base in the overlapping region, to obtain the single denoised variants. After chimera removal, the final amplicon sequence variants (ASVs) were mapped onto the Homo sapiens genome (assembly GRCh38.p13), using Bowtie (v2.3.5.1), in order to remove artefactual reads from the host. The Silva database (v138) was set as a reference to assign taxonomy to each ASV. Genus classification was achieved using the DADA2 naive Bayesian classifier method. The ASVs with an assigned genus but without exact species, were aligned using the Blastn tool (v2.10.0+) against the Silva database with a minimum of 97% of identity.

The relative abundance of bacterial genera and species could be computed for graphing and further analysis.

### SMDI score

The Subgingival Microbial Dysbiosis Index (SMDI) was applied for each sample as described by Chen, Marsh and Al-Hebshi^40^ and Mazurel et al.^38^,. In brief, read counts tables were generated at the species level and normalized by centered log-ratio (CLR) transformation. Discriminating species (DS) were those distinguishing health and periodontitis in the reference dataset, identified by mean decrease in Gini (MDG) values from random forest analysis. Based on a list of DS compiled at the 0.4 cut-off, which is a measure of the importance of a taxon to the classification, and their mean CLR abundances in health and periodontitis as determined in the study by Chen, Marsh and Al-Hebshi^40^, a SMDI score was calculated for each inoculum or biofilm sample as follows: SMDI = mean CLR abundance of dysbiotic DS - mean CLR abundance of “normobiotic” (i.e., the composition in health) DS.

### Statistical analysis

Statistical analyses were performed in IBM SPSS statistics (version 29.0.1.0) with statistical significance set at *p* < 0.05, and GraphPad Prism (version 10.4.0) was used to create the figures. The Shapiro-Wilk test and visual inspections of histograms and Q-Q plots were used to assess the distribution of the data. Thereafter, real-time *in vitro* biofilm growth of subgingival plaque samples, pH, NO_3_^−^ and NO_2_^−^ concentrations in sample supernatants, DNA quantification, and analysis of the SDMI, the Shannon Diversity index and the chao1 index was conducted using repeated-measures ANOVA for normally distributed data or a Friedman test when the data was non-normally distributed. Post-hoc pairwise comparisons were conducted when a significant main effect was detected, using Bonferroni-corrected t-tests for parametric data (correction implemented in SPSS) or Wilcoxon signed-rank tests with Bonferroni correction for non-parametric data (correction manually applied). Normally distributed variables are presented as mean ± standard deviation (SD) and non-normally distributed variables are presented as median and interquartile range (IQR).

Only genera and species with a minimal detection signal were included in the microbiome statistical analysis. Specifically, a species or genus was considered if it was present in at least 60% of the samples from one of the eight conditions, with an abundance exceeding ten times the smallest percentage above zero.

The relative abundances of species and genera were standardized using ANCOM-BC and compared using a Wilcoxon signed rank tests (i.e., wilcox.test function of stats library of R) as previously described by Rosier et al.^26^, and considered statistically significant at an adjusted p-value < 0.05. Other statistical analyses of the bacterial microbiome data were performed according to Johnston et al.^41^, using R. Relative abundances were used to calculate alpha diversity (i.e., Shannon and Chao1 indexes), while ANCOM-BC-transformed abundances were used for Adonis tests (Permutational Multivariate Analysis of Variance Using Distance Matrices) and visualization of bacterial composition in a two-dimensional map using constrained correspondence analysis (CCA). All analyses were performed using the R Vegan library^42^.

## Results

### Effects of each test condition on biofilm growth and microbial metabolism (nitrate, nitrite and pH)

**Fig. 1:**
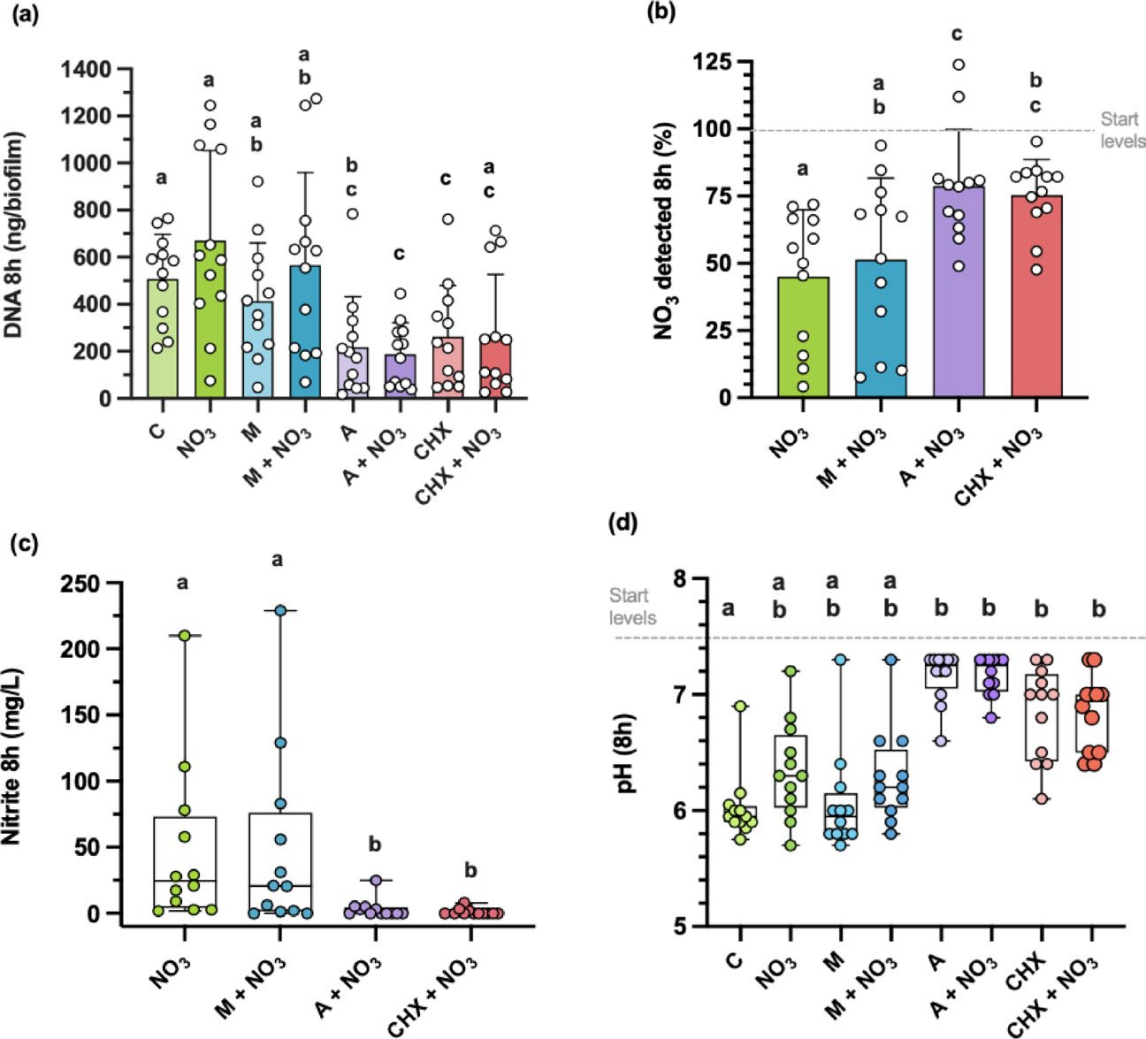
Effect of each treatment condition on biofilm growth and microbial metabolism after 8 h. **(a)** DNA quantification of the biofilms harvested at 8 h, across each test condition (a proxy for biofilm mass). Error bars show the mean ± SD of twelve donors **(b)** NO_3_^−^ reduction (% detected), with error bars showing the mean ± SD of twelve donors **(c)** NO_2_^−^ production (mg/L), with box-and-whisker plots showing the median and IQR of twelve donors **(d)** Effects of each treatment condition on pH, with box-and-whisker plots showing the median and IQR of twelve donors. All pairwise comparisons were Bonferroni-adjusted. For repeated-measures ANOVA (panels a and b), SPSS provided the adjusted p-values, with significance interpreted at *p* ≤ 0.05. For Friedman tests (panels c and d), Bonferroni corrections were applied manually, with adjusted significance thresholds of (c) *p* ≤ 0.008 (d) and *p* ≤ 0.002. Individual points represent one donor and conditions sharing the same letter are non-significant. C: control, NO_3_^−^: nitrate, CHX: chlorhexidine, M: metronidazole, A: amoxicillin.

Biofilm development was determined by the quantification of DNA (a proxy for biofilm biomass) after 8 h of growth (Fig. 1a). Biofilm biomass was ≈ 50% higher in the control condition compared to amoxicillin alone, amoxicillin + NO_3_^−^ (both, *p* < 0.001), and chlorhexidine alone (*p* = 0.04). Additionally, biofilm biomass was also ≈ 75% higher in the NO_3_^−^ condition compared to amoxicillin alone, chlorhexidine alone (both, *p* = 0.01), and amoxicillin + NO_3_^−^ (*p* = 0.005). Lastly, biofilm biomass was also significantly higher in the metronidazole condition compared to amoxicillin + NO_3_^−^ (*p* = 0.04) and chlorhexidine alone (*p* = 0.005) and was also ≈ 50% higher in the metronidazole + NO_3_^−^ condition compared to amoxicillin + NO_3_^−^ and chlorhexidine alone (both, *p* = 0.04). Interestingly, no significant differences in biofilm biomass were observed between the control condition, NO_3_^−^, metronidazole alone, and metronidazole + NO_3_^−^ (all, *p* > 0.05). There were also no statistically significant differences between amoxicillin, amoxicillin + NO_3_^−^, chlorhexidine alone and chlorhexidine + NO_3_^−^ (all, *p* = 1).

The nitrate reduction capacity (NRC) of the *in vitro* communities was determined in the supernatant of all of samples grown with NO_3_^−^ after 8 h of incubation by measuring NO_3_^−^ and NO_2_^−^ concentrations (Fig. 1b and c, respectively). Overall, the results showed that (%) NO_3_^−^ was ≈ 50% lower in the NO_3_^−^ condition compared to amoxicillin + NO_3_^−^ and chlorhexidine + NO_3_^−^ (both, *p* = 0.005). Similarly, (%) NO_3_^−^ was also significantly lower in the metronidazole + NO_3_^−^ condition compared to the amoxicillin + NO_3_^−^ condition (*p* = 0.02). In contrast, no significant difference in (%) NO_3_^−^ was found between NO_3_^−^ and metronidazole + NO_3_^−^ (*p* = 0.42), or between the amoxicillin + NO_3_^−^ and the chlorhexidine + NO_3_^−^ conditions (*p* = 1).

In agreement with the NRC results, NO_2_^−^ levels (mg/L) were significantly lower in the amoxicillin + NO_3_^−^ condition (*r* = −1.2, *p* = 0.003) and the chlorhexidine + NO_3_^−^ condition (*r* = −1.3, *p* = 0.002) compared with the NO_3_^−^ alone condition. NO_2_^−^ levels were also significantly lower in in the amoxicillin + NO_3_^−^ condition (*r* = −1.1, *p* = 0.007) and the chlorhexidine + NO_3_^−^ condition (*r* = −1.1, *p* = 0.005) compared to the metronidazole + NO_3_^−^ condition. In contrast, there was no significant differences detected between metronidazole + NO_3_^−^ and NO_3_^−^ alone (*r* = −0.3, *p* = 0.48), or between the chlorhexidine + NO_3_^−^ and amoxicillin + NO_3_^−^ conditions (*r* = − 0.6, *p* = 0.18) (Bonferroni adjusted p value, ≤ 0.008).

Finally, the effect of each treatment condition on pH was analysed (Fig. 1d). pH levels were significantly lower in the control condition compared to the amoxicillin, amoxicillin + NO_3_^−^, chlorhexidine and chlorhexidine + NO_3_^−^ conditions (all, *r* = − 0.6, *p* = 0.002). No significant differences were observed between NO_3_^−^ alone and the metronidazole + NO_3_^−^ condition (*r* = − 0.3, *p* = 0.1). Additionally, no significant differences were detected between chlorhexidine alone and amoxicillin alone, or the chlorhexidine + NO_3_^−^ condition and the amoxicillin + NO_3_^−^ condition (both, *r* = −0.5 and *p* > 0.002 (Bonferroni adjusted p value, ≤ 0.002).

### Effects of each test condition on biofilm composition

At the species level, a total of 7,849,612 raw reads were obtained across all samples (mean = 72,682 reads per sample). After quality filtering, 7,424,112 reads were retained (mean = 68,742 reads per sample), corresponding to 94.6% of the original sequences. At the genus level, the same number of raw reads were obtained (7,849,612; mean = 72,682 reads per sample), and after filtering, 7,690,924 reads remained (mean = 71,212 reads per sample), representing 98.0% of the total reads. The filtered abundance tables at both the species and genus levels were used for downstream community analyses.

**Fig. 2:**
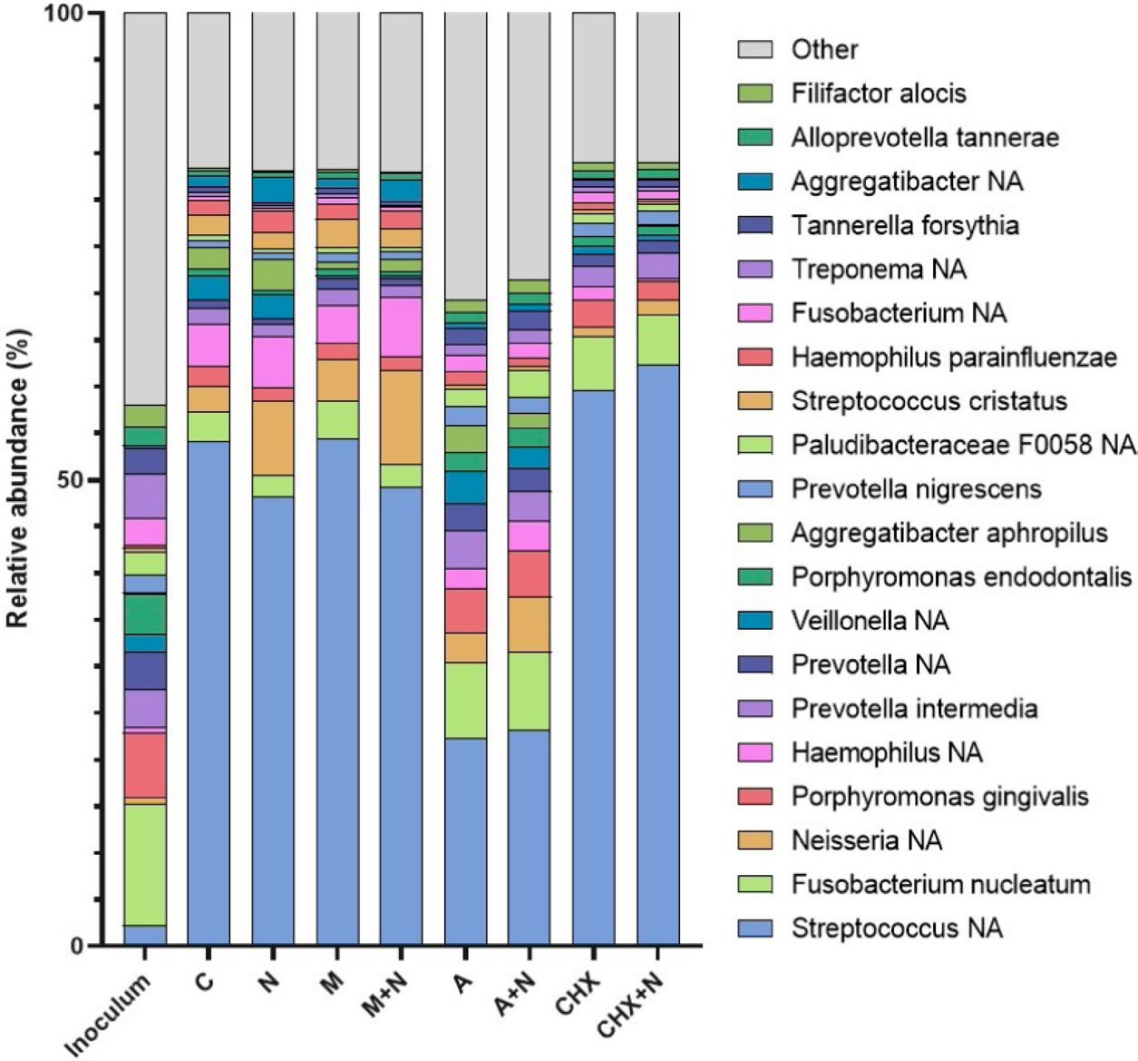
Effect of each test condition on *in vitro* biofilm composition after 8 h growth. Periodontal plaque samples grown in 8mM NO_3_^−^, metronidazole, amoxicillin and chlorhexidine (both with and without 8mM of NO_3_^−^) are compared with the initial inoculum and untreated control (*n* = 12). Relative abundance of species at the taxonomic level, determined by 16 s rRNA gene sequencing. The top 20 most abundant species for each test condition are shown and sorted by overall mean abundance (all other species are shown as “other”). C: control, NO_3_^−^: nitrate, CHX: chlorhexidine, M: metronidazole, A: amoxicillin.

The effect of each treatment on biofilm composition after 8 h of growth was analysed by 16 s rRNA gene analysis (Fig. 2). Firstly, the most dominant taxa in the inoculum (subgingival plaque), included *Fusobacterium nucleatum*,* Porphyromonas gingivalis*,* Treponema* NA, *Porphyromonas endodontalis* and *Prevotella* NA. This composition is typical of periodontitis-associated plaque samples and reflects their clinical origin^38^. In terms of the control condition, *Streptococcus* NA, *Haemophilus* NA, *Fusobacterium nucleatum*,* Veillonella* NA and *Neisseria* NA were the most dominant taxa, whereas *Streptococcus* NA, *Neisseria* NA, *Haemophilus* NA, *Aggregatibacter aphrophilus* and *Aggregatibacter* NA dominated the NO_3_^−^ condition. *Streptococcus* NA, *Neisseria* NA, *Fusobacterium nucleatum*,* Haemophilus* NA and *Streptococcus cristatus* were predominant in the metronidazole condition, compared with *Streptococcus* NA, *Neisseria* NA, *Haemophilus* NA, *Aggregatibacter* NA and *Fusobacterium nucleatum* in the metronidazole + NO_3_^−^ samples. Additionally, the main taxa present in the amoxicillin condition included *Streptococcus* NA, *Fusobacterium nucleatum*,* Porphyromonas gingivalis*,* Prevotella intermedia* and *Veillonella* NA, whereas *Streptococcus* NA, *Fusobacterium nucleatum*,* Neisseria* NA, *Porphyromonas gingivalis* and *Prevotella intermedia* dominated the amoxicillin + NO_3_^−^ condition. Lastly, the chlorhexidine and chlorhexidine + NO_3_^−^ conditions were dominated by similar taxa, including *Streptococcus* NA, *Fusobacterium nucleatum*,* Porphyromonas gingivalis*,* Prevotella intermedia* and *Prevotella nigrescens.*

**Fig. 3:**
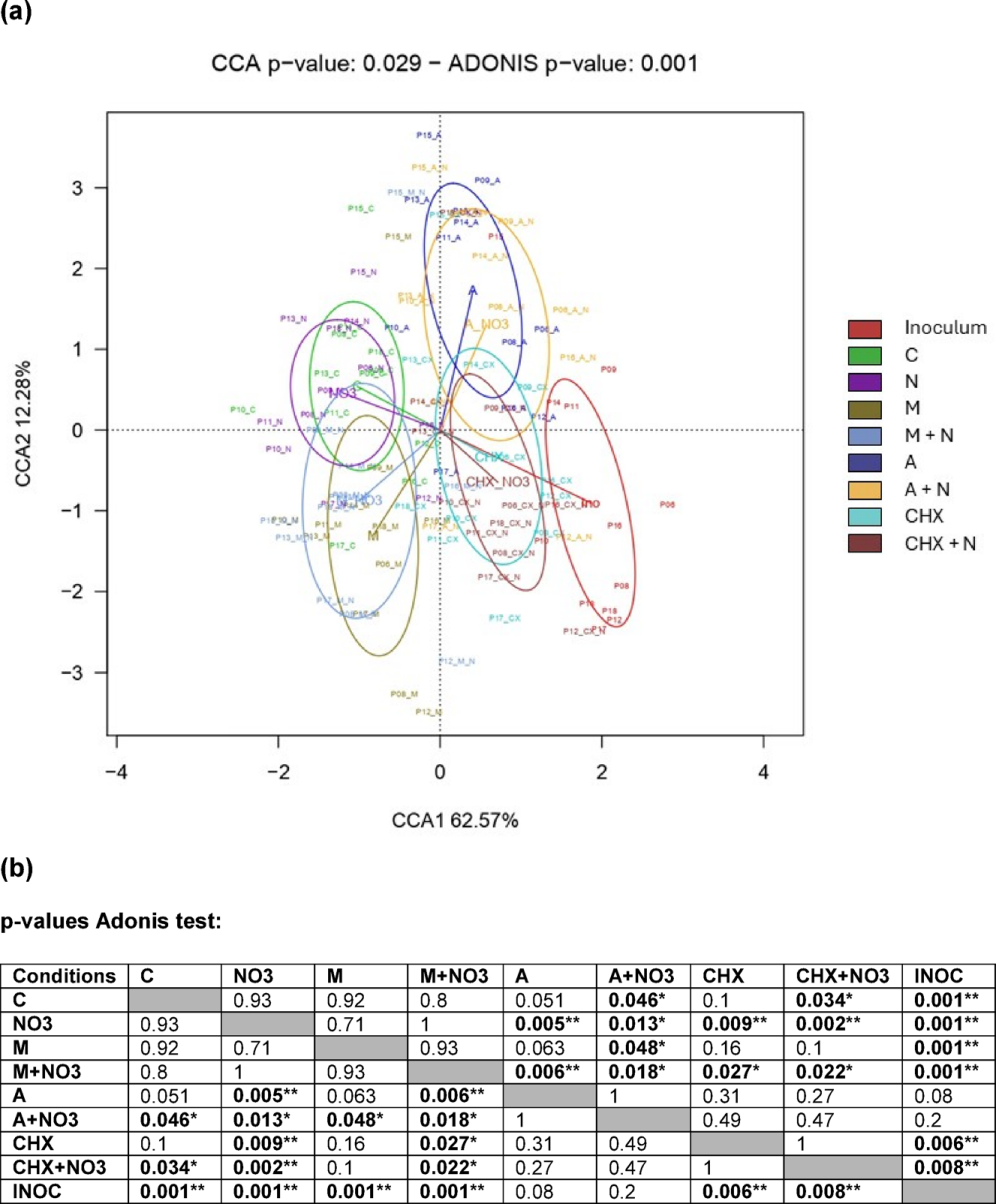
Effect of each treatment condition on oral biofilm composition (beta-diversity) after 8 h of *in vitro* growth. **(a)** Canonical Correspondence Analysis (CCA) of microbial community composition across treatments at the species level. The first two axes explain 62.57% and 12.28% of the constrained variance, respectively. Significance was assessed using 999 permutations (CCA *p* = 0.029; Adonis *p* = 0.001). **(b)** Adonis test p-values. Numbers in bold indicate significance (*p < 0.05, ** *p* < 0.01). C: control, NO_3_^−^: nitrate, CHX: chlorhexidine, M: metronidazole, A: amoxicillin.

The beta-diversity of the bacterial communities was represented in a canonical correlations analysis (CCA) plot (Fig. 3a), and significant differences were determined with an Adonis test (Fig. 3b). Differences in beta-diversity were found between the control condition and chlorhexidine + NO_3_^−^ condition (*p* < 0.05), between the control condition and amoxicillin condition (*p* = 0.051), and between the control condition and amoxicillin + NO_3_^−^ condition (*p* < 0.05). Additionally, differences in beta-diversity were found in the control, NO_3_^−^, metronidazole, metronidazole + NO_3_^−^, chlorhexidine and chlorhexidine + NO_3_^−^ conditions compared with the inoculum (*p* < 0.01). However, amoxicillin alone and the amoxicillin + NO_3_^−^ condition did not differ significantly from the inoculum, indicating bacterial growth could be limited.

**Fig. 4:**
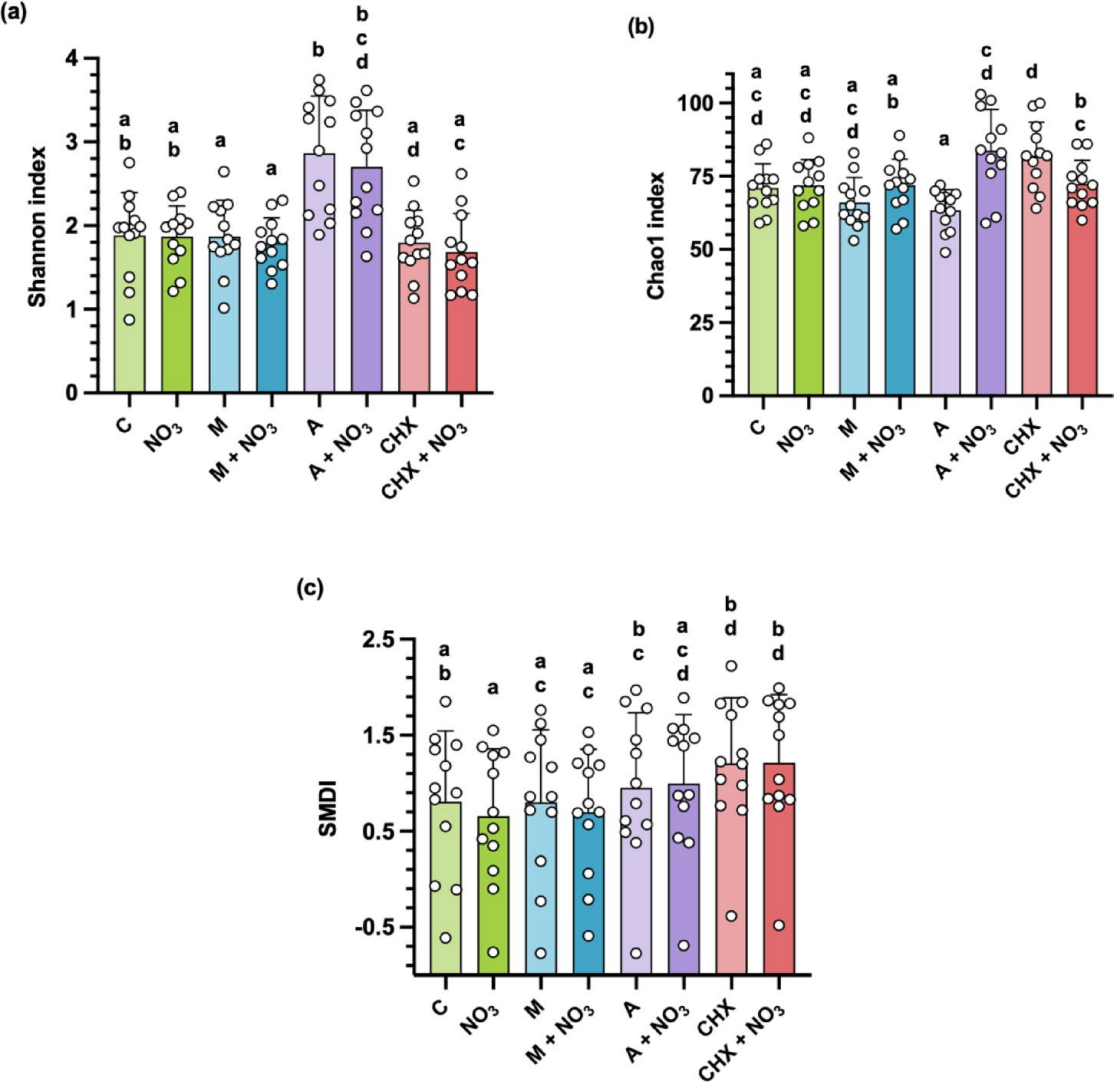
Biofilm composition and microbial diversity post-treatment (alpha-diversity). **(a)** Shannon index **(b)** Chao1 index **(c)** Subgingival microbial dysbiosis index. Individual points represent one donor and error bars show the mean ± SD of twelve donors. Pairwise comparisons were performed following repeated-measures ANOVA, with Bonferroni-adjusted p-values provided by SPSS; significance was interpreted at *p* ≤ 0.05. Conditions sharing the same letter are non-significant. C: control, NO_3_^−^: nitrate, CHX: chlorhexidine, M: metronidazole, A: amoxicillin.

Looking at alpha-diversity and the SMDI dysbiosis index, none of the conditions significantly affected alpha-diversity or the SMDI dysbiosis index when compared with the control condition (Fig. 4a and c) (*p* > 0.05). However, a lower SMDI dysbiosis index was found in the NO_3_^−^ condition compared with amoxicillin alone, chlorhexidine alone (both, *p* < 0.05) and the chlorhexidine + NO_3_^−^ condition (*p* < 0.001) (Fig. 4c). Similarly, a lower SMDI dysbiosis index was found in the metronidazole alone and the metronidazole + NO_3_^−^ conditions compared with the chlorhexidine condition and the chlorhexidine + NO_3_^−^ condition (all, *p* < 0.05).

**Fig. 5:**
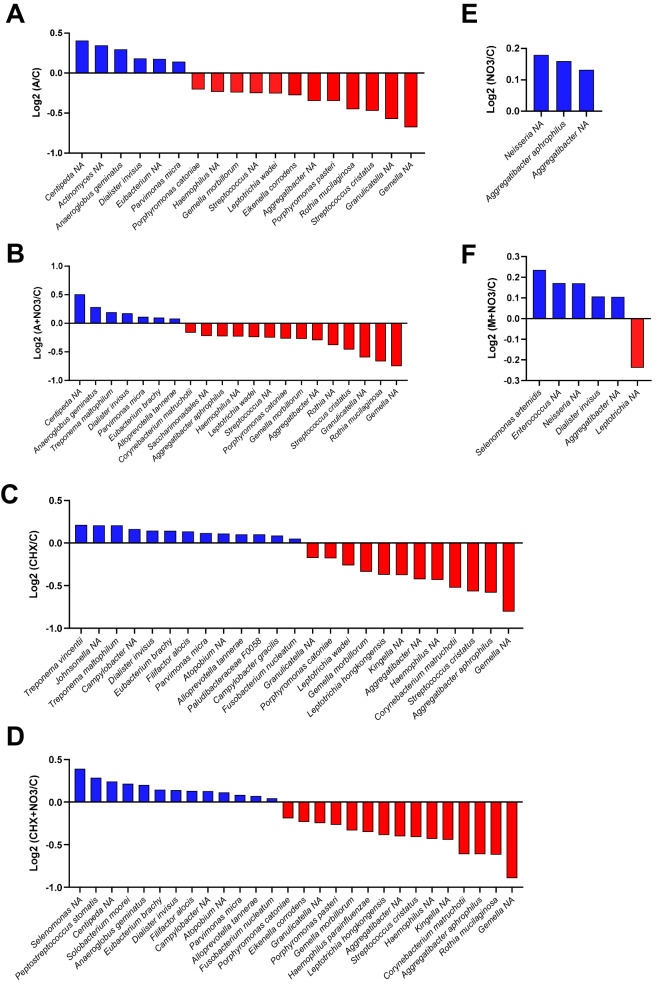
The effects of each test condition on biofilm composition. Significant changes are expressed as Log_2_ fold-changes of relative abundance of all species (*n =* 12). **(a)** A/C: Amoxicillin vs. control **(b)** A + NO3/C: Amoxicillin + NO_3_^−^ vs. control **(c)** CHX/C: Chlorhexidine vs. control **(d)** CHX + NO3/C: Chlorhexidine + NO_3_^−^ vs. control **(e)** NO3/C: NO_3_^−^ vs. control **(f)** M + NO3/C: Metronidazole + NO_3_^−^ vs. control. Blue = increase; Red = Decrease. Each panel represents a separate comparison against the control.

At the individual species level, significant changes (*p* < 0.05) were observed between the control and the amoxicillin, amoxicillin + NO_3_^−^, chlorhexidine, chlorhexidine + NO_3_^−^, NO_3_^−^, and metronidazole + NO_3_^−^ conditions (Fig. 5a and f). Generally, disease associated species increased whilst health-associated species decreased in the amoxicillin and amoxicillin + NO_3_^−^ conditions compared with the control condition (Fig. 5a and b). For instance, the abundance of *Centipeda* NA, *Treponema* NA, *Dialister invisus*,* Parvimonas micra* and *Eubacterium brachy* increased, whilst *Gemella* NA and *Rothia mucilaginosa* decreased. Similarly, the chlorhexidine and chlorhexidine + NO_3_^−^ conditions saw a decrease in the abundance of health-associated species, including *Gemella* NA, *Rothia* NA, *Kingella* NA and *Haemophilus* NA, whilst an increase in disease-associated species, such as *Fusobacterium nucleatum*,* Treponema vincentii*,* Eubacterium brachy* and *Dialister invisus*, was observed (Fig. 5c and d). In contrast, the NO_3_^−^ condition saw an increase in the abundance of health-associated species, such as *Neisseria* NA and *Aggregatibacter* NA, whilst the metronidazole + NO_3_^−^ condition saw both an increase in disease-associated species, including *Dialister invisus*, and health associated species, like *Neisseria* NA, compared with the control condition (Fig. 5e and f).

**Fig. 6:**
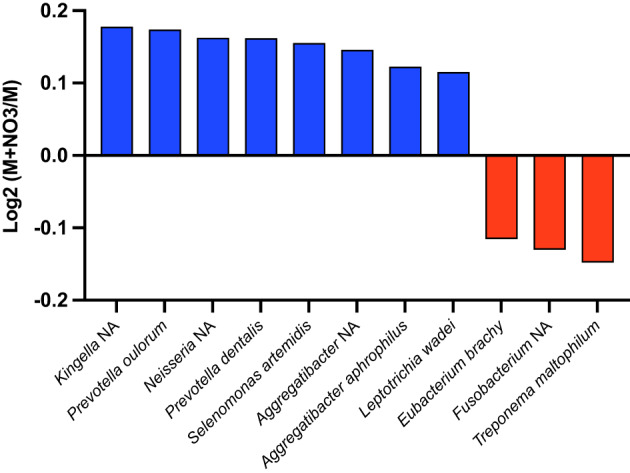
The effect of adding NO_3_^−^ to metronidazole vs. metronidazole alone. Significant changes in biofilm composition are expressed as Log_2_ fold-change of relative abundance of all species (*n =* 12). Blue = increase; Red = Decrease.

Moreover, the addition of NO_3_^−^ to metronidazole led to statistically significant increases (*p* < 0.05) in health-associated nitrate-reducing *Neisseria* NA and *Kingella* NA species, and a reduction in three periodontal disease-associated bacteria, namely *Fusobacterium* NA, *Eubacterium brachy* and *Treponema maltophilum* (Fig. 6). When comparing amoxicillin with the amoxicillin + NO_3_^−^ condition, and chlorhexidine with the chlorhexidine + NO_3_^−^ condition, no significant changes were found (*p* > 0.05).

## Discussion

In this study, we aimed to determine the effects of amoxicillin, metronidazole and chlorhexidine, in the presence or absence of NO_3_^−^, on the bacterial composition of subgingival plaque of periodontitis patients and assess the effects of these compounds on NO_3_^−^ metabolism. For this, chlorhexidine and amoxicillin had to be diluted to sublethal concentrations (0.002% and 0.7 µg/ml, respectively), while metronidazole could be studies at physiologically relevant GCF levels (16 µg/ml). Across all conditions, excluding amoxicillin and amoxicillin + NO_3_^−^, the overall bacterial composition of the biofilms after 8 h of growth was significantly different from the inoculum, which contained planktonic cells and represented the initial dysbiotic state of the plaque samples. More specifically, our data showed that adding metronidazole (16 µg/ml) to subgingival plaque did not inhibit bacterial growth or NO_3_^−^ metabolism under the in vitro conditions used. However, the addition of NO_3_^−^ to metronidazole (compared with metronidazole alone) led to decreases in disease-associated bacteria, including *Fusobacterium* spp., *Treponema maltophilum* and *Eubacterium brachy*, whilst levels of health-associated *Kingella* and *Neisseria* spp. increased. In contrast, chlorhexidine (0.002%) limited biofilm growth, bacterial metabolism and NO_3_^−^ reduction, and increased dysbiosis. Samples treated with chlorhexidine or chlorhexidine + NO_3_^−^ saw significant increases in periodontitis-associated bacteria, including *Fusobacterium nucleatum*,* Dialister invisus*,* Eubacterium brachy*,* Filifactor alocis*,* Parvimonas micra*,* Alloprevotella tannerae* and *Treponema vincentii.* Lastly, we found that low concentrations of amoxicillin (0.7 µg/ml) limited bacterial growth, microbial metabolism and NO_3_^−^ reduction. The subgingival plaque samples treated with amoxicillin appeared to maintain a similar overall bacterial composition to the inoculum, indicating that *in vitro* growth was limited compared with the control condition. This was accompanied by disease-associated changes in bacterial species in the amoxicillin condition.

Metronidazole and amoxicillin are commonly used as mono- or dual-therapies in conjunction with mechanical plaque removal to treat periodontal disease, with the main aim of eradicating pathogenic bacteria and reducing the hosts inflammatory response^10,43–45^. In short, metronidazole inhibits DNA synthesis inside bacterial cells, whilst amoxicillin inhibits cell wall synthesis, both causing cell death^10,46^. Several studies have shown that combining professional mechanical plaque removal (PMPR) with metronidazole + amoxicillin can significantly improve clinical attachment level (CAL) and reduce periodontal pocket depth (PPD) in disease subjects^43–46^. Additionally, metronidazole + amoxicillin also significantly improved clinical outcomes (PPD and CAL) in patients with severe and chronic periodontitis^47–49^, and *in vitro* studies have reported a reduction or eradication of periodontal disease-associated pathogens when oral biofilms were exposed to both antibiotics, thus including *Porphyromonas gingivalis* and *Fusobacterium nucleatum*, although these effects may differ for complex biofilms derived from oral samples^50,51^. Unfortunately, however, although antibiotics are traditionally used to treat periodontitis, an overreliance on these compounds is contributing to the global burden of antimicrobial resistance^52,53^.

In this study, subgingival plaque samples treated with low levels of amoxicillin (0.7 µg/ml) limited bacterial growth and NO_3_^−^ reduction, which is consistent with its non-selective, broad-spectrum activity against aerobic and facultative anaerobic species. It is likely that, due to its non-selective activity, amoxicillin did not reduce dysbiosis, which suggests that this antibiotic can suppress microbial growth and activity without restoring community balance. In fact, amoxicillin led to several disease-associated changes, such as an increase in *Treponema maltophilum* and a decrease in *Rothia mucilaginosa*, compared with the control condition. Some of these differences may have stemmed from limited in vitro growth in the presence of amoxicillin, which preserved the initial dysbiosis of the inoculum, whereas others may have reflected differences in species-specific amoxicillin sensitivity.

On the other hand, metronidazole (16 µg/ml) did not limit bacterial growth, which may have resulted from the in vitro conditions of this experiment limiting the activation of this prodrug. After activation by bacterial enzymes, metronidazole targets obligate anaerobes (which are mostly associated with periodontitis), while facultative anaerobes may lack the enzymes required to activate this antibiotic^46,54^. Regarding this, streptococci had a selective advantage in non-selective BHI medium used in our study, which has also been observed in other in vitro studies using complex oral samples and common growth media^16,38,55^. Future studies should consider using media specifically developed to support the growth of subgingival or periodontal plaque communities, such as the recently introduced serum-containing medium described by Baraniya et al.^56^.

Interestingly, in our study, metronidazole alone did not alter health- or disease- associated bacteria, whereas the combination of metronidazole + NO_3_^−^ resulted in different changes compared with the control condition, including an increase in health-associated *Neisseria* spp. and a decrease in *Leptotrichia* spp. This could have resulted from more metronidazole activation in the presence of NO_3_^−^, which should be investigated in future studies. Regarding these findings, previous *in vitro* studies have shown that metronidazole is most effective when used in synergy with other antibiotics^50,57^. Notably, metronidazole and amoxicillin are often combined as a treatment in periodontics^46,47^. We analysed the effects of amoxicillin and metronidazole separately to allow us to identify the individual effects of each antibiotic on bacterial growth, NO_3_^−^ metabolism and community composition. However, future studies should investigate the effects of the dual antibiotic treatment.

Importantly, multiple clinical studies have shown that metronidazole alone can reduce pathogenic bacteria in periodontitis patients^58,59^, thus, potentially due to the drugs anti-inflammatory properties causing a reduction in inflamophilic species^60,61^. In our study, in which we tested the effects of compounds on the subgingival microbiota without the influence of the immune system, we found that adding NO_3_^−^ to metronidazole (compared with metronidazole alone) increased health-associated *Neisseria and Kingella* species, and decreased periodontal disease-associated pathogens, including *Eubacterium brachy*,* Treponema maltophilum* and *Fusobacterium* spp. This indicates that NO_3_^−^ metabolism could have stimulated the activation of metronidazole and vice versa, which warrants further investigation. Importantly, in one study, one week of NO_3_^−^ rich lettuce juice reduced gingival inflammation compared with a NO_3_^−^ depleted lettuce juice placebo^15^. Future clinical studies should therefore explore if combining NO_3_^−^ with metronidazole leads to synergistic effects that improve clinical outcomes. In this study, the SMDI dysbiosis index was significantly lower in the metronidazole + NO_3_^−^ and NO_3_^−^ alone conditions compared with chlorhexidine alone and the chlorhexidine + NO_3_^−^ condition. In addition, NO_3_^−^ alone also had a lower SMDI than the amoxicillin alone condition but not compared with the amoxicillin + NO_3_^−^ condition. These outcomes could result from SMDI lowering effects of NO_3_^−^, as previously observed Mazurel et al.^38^, and/or potential SMDI increasing effects of amoxicillin and chlorhexidine in some individuals (i.e., the average SMDI of conditions with amoxicillin and chlorhexidine was higher than in the control condition but this was not significant).

This study showed that low concentrations of chlorhexidine (0.002%) limited biofilm growth, bacteria metabolism and NO_3_^−^ reduction, whilst increasing the abundances of some disease-associated species, including *Fusobacterium nucleatum*,* Dialister invisus* and *Treponema vincentii.* Moreover, beta-diversity and the SMDI dysbiosis index were significantly different in the chlorhexidine alone and chlorhexidine + NO_3_^−^ conditions compared with NO_3_^−^ alone and the metronidazole + NO_3_^−^ conditions. Our findings are consistent with previous studies that showed chlorhexidine can impair NO_3_^−^ reduction^13,25^. However, chlorhexidine does have anti-plaque and anti-inflammatory properties^10,62^. The compound is cationic and particularly targets gram-positive bacteria, causing damage to bacterial cell membranes, which results in cell death^10^. Several studies have highlighted the benefits of chlorhexidine when used as an adjunct therapy to PMPR to treat periodontitis. Chlorhexidine chips and gels have been shown to improve clinical outcomes, such as PPD and CAL, as well as reducing the plaque index (PI) and gingival index (GI) in disease patients^63–65^. Chlorhexidine mouthwashes (0.06% − 0.2%) are also used during orthodontic treatment and after periodontal or implant surgery to control plaque and reduce gingival inflammation^66–68^.

In contrast, evidence suggests that chlorhexidine can have detrimental effects, even at low concentrations^62^. These mouthwashes and gels can damage the oral mucosa, alter taste and cause tooth discolouration^10,12,62^. Moreover, as chlorhexidine is non-selective, studies have shown that this compound can disrupt the oral microbiome and/or increase oral acidity levels, which increases the risk of caries^10,13^. Additionally, chlorhexidine diffusion through thick subgingival biofilms can be reduced, and bacterial exposure to sub-lethal concentrations increases the risk of AMR^10,62^. Lastly, recent studies have shown that chlorhexidine resistance in oral bacteria can also lead to cross-resistance to antibiotics^69^, including tetracycline, ampicillin, clindamycin and erythromycin^14,70^. Based on our findings and previous results, a secondary effect of chlorhexidine could include a disruption of the microbial community towards oral diseases and the loss of NO_3_^−^ reduction, which is important for oral and systemic health. Thus, although chlorhexidine can be considered an effective antiseptic for disease prevention and improving periodontal outcomes, its undesirable side effects suggest that alternative treatment approaches should be explored.

Overall, we showed that 0.002% chlorhexidine, a concentration well below the therapeutic range of 0.06% − 0.12%, limited biofilm growth, bacterial metabolism and NO_3_^−^ reduction whilst increasing dysbiosis in subgingival plaque samples. The non-selective action of chlorhexidine, combined with its stronger effect on Gram-positive bacteria, likely contributed to microbial community shifts that favoured disease-associated Gram-negative species, despite the overall reductions observed in growth and metabolism. Future studies should investigate whether microbial regrowth following chlorhexidine treatment maintains the increased dysbiosis observed in this study. Our findings complement studies which have found both that chlorhexidine can impair NO_3_^−^ metabolism^13,25^, and that this compound is most effective against gram-positive bacterial species^10,71,72^. Thus, in addition to its adverse side effects, long-term chlorhexidine treatment may be counterproductive in managing periodontitis, as the disease is primarily associated with gram-negative anaerobes^10^. Prolonged or frequent use of chlorhexidine could, therefore, promote increased resistance and a higher relative abundance of gram-negative bacteria in subgingival plaque, which should be confirmed in clinical studies.

We found that the SMDI dysbiosis index was lower in the NO_3_^−^ (8 mM) condition compared to amoxicillin alone and chlorhexidine with or without NO_3_^−^. Compared to the control condition, NO_3_^−^ led to beneficial increases in health-associated *Neisseria* species. Additionally, an increase in unclassified *Aggregatibacter* species was observed. However, the periodontitis-associated *A. actinomycetemcomitans*, which was detected separately, did not increase and has previously been shown to decrease in the presence of NO_3_^−^^38^. Further research is needed to identify which *Aggregatibacter* species may increase in response to NO_3_^−^, as this genus also includes health-associated members^73^. Similarly, when adding NO_3_^−^ to metronidazole (compared to metronidazole alone), nitrate-reducing *Kingella* and *Neisseria* species increased, while periodontitis-associated species (e.g., *Fusobacterium* spp., *Treponema maltophilum* and *Eubacterium brachy*) decreased. Our findings are consistent with other studies that have reported an increase in NRB associated with eubiosis, such as *Rothia* and *Neisseria* spp., and a decrease in bacteria associated with dysbiosis, including *Porphyromonas*,* Fusobacterium* and *Prevotella* spp., following NO_3_^−^ supplementation^13,16,74,75^. Moreover, studies have also shown that NO_3_^−^ can lower oral acidity levels, which reduces the risk of dental caries^16,74,76,77^. Thus, it is already well documented in the literature that NO_3_^−^ promotes eubiosis in individuals without periodontitis. Regarding this, studies have also shown that NO_3_^−^ can reduce dysbiosis and gingival inflammation in individuals with gingivitis, potentially limiting progression to periodontitis^15,28,38^. Results from this work support these findings and demonstrated that treating dysbiotic subgingival plaque samples with NO_3_^−^ alone could reduce dysbiosis compared with commonly used antimicrobial compounds, and promote eubiosis by increasing health-associated *Neisseria* spp.

Importantly, this study supports the idea that NO_3_^−^, possibly in combination with metronidazole, should be further explored as a therapeutic intervention for periodontal diseases. Both NO_3_^−^^15^ and metronidazole^60^ by themselves have anti-inflammatory effects, and their potential synergy should be further studied in clinical studies. By reducing inflammation, the growth of inflamophilic species is decreased, which can further improve the composition of the subgingival microbiota. Combining NO_3_^−^ with a short-term regimen or a low concentration of metronidazole could help limit antibiotic exposure, but clinical studies are needed to confirm this hypothesis.

The limitations of this study included a small number of donors, leading to a total *n =* 12 individuals. However, in previous studies in which complex oral samples of 10–12 individuals were included, significant results were also obtained^16,38^. Additionally, in the current *in vitro* system, no host cells were added. On the one hand, this means that the activity of the host immune system, which affects the subgingival plaque composition, was not considered. On the other hand, by focusing only on the microbial community, the ecological effect of the different compounds can be determined. Another limitation of the study was that very low concentrations of amoxicillin (0.7 µg/ml) appeared to limit changes in the community structure and growth compared with the inoculum. Even lower concentrations could help to determine the exact ecological effects on the microbial community in future in vitro studies. Finally, even the nonspecific BHI medium seemed to favour streptococci, suggesting that future studies should test alternative media to reduce this selective effect.

In conclusion, findings from this study show that bacterial growth, bacterial metabolism and NO_3_^−^ reduction were limited by low concentrations of chlorhexidine and amoxicillin, but not physiologically relevant concentrations of metronidazole. Adding NO_3_^−^ to metronidazole led to health-associated changes in bacterial composition compared with metronidazole alone. In contrast, conditions with chlorhexidine and amoxicillin were associated with more disease-related bacterial profiles. Additionally, subgingival plaque treated with NO_3_^−^ alone saw increases in health-associated *Neisseria* spp. Findings from this study suggest that chlorhexidine and amoxicillin may have additional side-effects beyond those already established, including potential increases in disease-associated bacteria in subgingival plaque communities and a negative impact on NO_3_^−^ metabolism; further studies are required to determine if these effects occur in vivo during and after treatment with these antimicrobial agents. Additionally, our results suggest that a dual treatment of metronidazole + NO_3_^−^ may have beneficial effects, which should be evaluated in clinical studies.

## Data Availability

All sequencing reads are deposited in the NCBI Sequencing Read Archive (SRA) under BioProject PRJNA1307442. Any further data is available upon reasonable request from the corresponding author.
